# Tetra­kis[*μ*-*N*,*N*′-bis­(4-bromo­phen­yl)formamidinato-κ^2^ *N*:*N*′]dimolyb­denum(II) tetra­hydro­furan solvate

**DOI:** 10.1107/S1600536811011202

**Published:** 2011-03-31

**Authors:** L.-J. Han

**Affiliations:** a Department of Chemistry, Tongji University, Shanghai 200092, People’s Republic of China

## Abstract

The title complex, [Mo_2_(C_13_H_9_N_2_Br_2_)_4_]·C_4_H_8_O, contains a quadruply bonded Mo_2_^4+^ unit equatorially coordinated by four *N*,*N′*-bis­(4-bromo­phen­yl)formamidinate ligands, forming a dimetal paddlewheel complex. The centroid of the Mo—Mo bond is located on a special position with *2/m* symmetry. In the crystal, complex mol­ecules are linked by Br⋯Br inter­actions [3.7049 (10) Å]. The disordered solvent mol­ecule could not be satisfactorily modelled and was therefore eliminated from the final refinement.

## Related literature

For the nature of halogen–halogen inter­actions, see: Domercq *et al.* (2001 ▶); Espallargas *et al.* (2006 ▶). For Br⋯Br inter­actions, see: Fujiwara *et al.* (2006 ▶); Reddy *et al.* (1996 ▶). For the use of inter­molecular inter­actions in supra­molecular synthesis, see: Brammer (2004 ▶); Desiraju (1995 ▶, 2001 ▶).
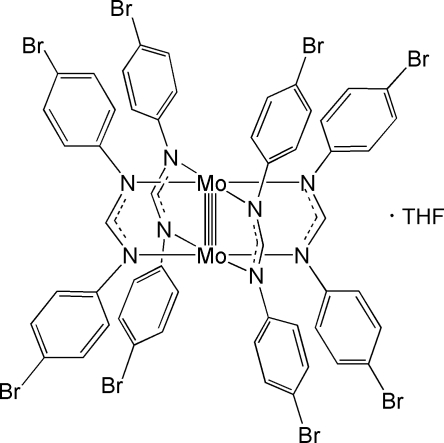


## Experimental

### 

#### Crystal data

[Mo_2_(C_13_H_9_N_2_Br_2_)_4_]·C_4_H_8_O
*M* *_r_* = 1604.05
Monoclinic, 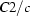

*a* = 21.795 (4) Å
*b* = 10.077 (2) Å
*c* = 29.967 (6) Å
β = 110.67 (3)°
*V* = 6158 (2) Å^3^
*Z* = 4
Mo *K*α radiation
μ = 5.64 mm^−1^
*T* = 293 K
0.15 × 0.13 × 0.10 mm

#### Data collection

BRUKER SMART 1000 diffractometer
Absorption correction: multi-scan (*SADABS*; Sheldrick, 2004 ▶) *T*_min_ = 0.429, *T*_max_ = 0.569
25098 measured reflections
5995 independent reflections
3563 reflections with *I* > 2σ(*I*)
*R*_int_ = 0.119

#### Refinement

*R*[*F* ^2^ > 2σ(*F* ^2^)] = 0.055
*wR*(*F* ^2^) = 0.151
*S* = 1.01
5995 reflections
317 parameters
H-atom parameters constrained
Δρ_max_ = 1.09 e Å^−3^
Δρ_min_ = −1.11 e Å^−3^

### 

Data collection: *APEX2* (Bruker, 2004 ▶); cell refinement: *SAINT-Plus* (Bruker, 2001 ▶); data reduction: *SAINT-Plus*; program(s) used to solve structure: *SHELXS97* (Sheldrick, 2008 ▶); program(s) used to refine structure: *SHELXL97* (Sheldrick, 2008 ▶); molecular graphics: *XP* (Sheldrick, 2008 ▶); software used to prepare material for publication: *SHELXL97* and *PLATON* (Spek, 2009 ▶).

## Supplementary Material

Crystal structure: contains datablocks global, I. DOI: 10.1107/S1600536811011202/ff2002sup1.cif

Structure factors: contains datablocks I. DOI: 10.1107/S1600536811011202/ff2002Isup2.hkl

Additional supplementary materials:  crystallographic information; 3D view; checkCIF report

## Figures and Tables

**Table 1:** Selected bond lengths (Å)

Mo1—Mo1^i^	2.1263 (13)
Mo1—N3	2.192 (5)
Mo1—N4^i^	2.195 (5)
Mo1—N1^i^	2.198 (5)
Mo1—N2	2.218 (5)

Symmetry code: (i) 
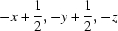
.

**Fig. 1.**
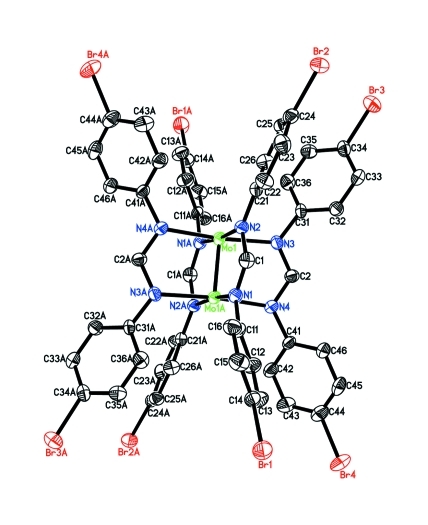
Molecular structure of the title compound drawn with displacement ellipsoids at the 30% probability level. All hydrogen atoms have been omitted for clarity. Atoms with suffix A are generated by the symmetry operation(-x + 1/2, -y + 1/2, -z).

**Fig. 2.**
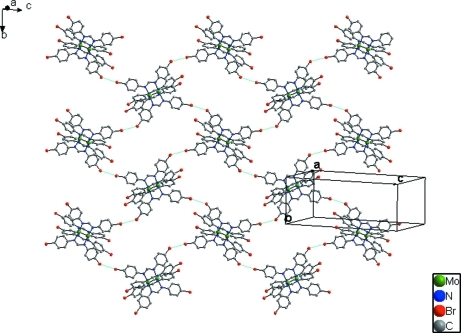
Part of a two-dimensional plane of the title compound. Br···Br interactions are drawn with blue dashed lines.

## supplementary crystallographic information

### Comment

One of the main interests of crystal engineering is the study of intermolecular interactions and their utilization in supramolecular synthesis (Desiraju 1995; Desiraju 2001; Brammer 2004). These interactions range from strong forces, *e.g.*, classical hydrogen bonds, to weaker ones, *e.g.*, halogen···halogen interactions. The nature of the halogen···halogen interactions has been studied both through extensive crystallographic investigation and using *ab initio* calculations(Domercq *et al.*,2001; Espallargas *et al.*, 2006). Here we report intermolecular Br···Br interactions in the crystal structure Mo_2_(C_13_H_9_N_2_Br_2_)_4_.THF.

The molecular structure of the title compound is shown in Fig.1. The molecule of Mo_2_(C_13_H_9_N_2_Br_2_)_4_ (I) occupies a special position on an inversion center, and the Mo—Mo distance is 2.1263 (13) Å, which is in the range of dimolybdenum quadruple bonds. Bromine atoms participate in short contacts (3.7049 (10) Å) linking the molecules of (I) into planes. This value is significantly shorter than van der Waals contact distance (3.90 Å) (Reddy *et al.*, 1996; Fujiwara *et al.*, 2006).

### Experimental

A mixture yellow Mo_2_(OOCCH_3_)_4_ (0.128 g, 0.300 mmol) and *N*,*N'*-bis(4-bromophenyl)formamidinate (0.425 g, 1.20 mmol) was suspended in 20 ml of THF. While stirring, 2.4 ml NaOCH_2_CH_3_ solution (0.5 *M* in ethanol) was added slowly. The colour turned first to red and then to dark red. The reaction was stirred for 5 h at room temperature, and then the volume of the solvent was reduced to about 3 ml under reduced pressure. The residue was washed with distilled water(3×10 ml) and ethanol (8 ml) and dried under vacuum. The yellow solid was dissolved in THF (15 ml) and the solution was layered with hexanes. Yellow block-shaped crystals formed after several days. Yield: 0.342 g (71%). ^1^HNMR(CDCl_3_, p.p.m.): 8.41(s, 4H, –NCHN–), 7.10(d, 16H, aromatic), 6.06(d, 16H, aromatic). Anal. Calcd. C_52_H_36_Mo_2_N_8_Br_8_: C, 38.94; H, 2.26; N, 6.99; Found: C, 38.78; H, 2.17; N, 7.08.

### Refinement

H atoms were positioned geometrically with C—H = 0.93, 0.97 and 0.96 Å, for aromatic, methylene, and methyl H atoms, respectively, and constrained to ride on their parent atoms, with *U*_iso_(H) = *xU*_eq_(C), where *x* = 1.5 for methyl H and *x* = 1.2 for aromatic H atoms.

A search for solvent-accessible voids in the crystal structure using *PLATON* (Spek, 2009) showed a potential solvent volume of 829.4 Å^3^ and subsequent application of SQUEEZE procedures showed four relevant voids each with a solvent-accessible volume of 207 Å^3^ . The SQUEEZE procedure was used to eliminate the contribution of the electron density in the solvent region from the intensity data, and the solvent-free model was employed in the final refinement.

### Crystal data

**Table:**

[Mo_2_(C_13_H_9_N_2_Br_2_)_4_]·C_4_H_8_O	*F*(000) = 3072
*M**_r_* = 1604.05	*D*_x_ = 1.730 Mg m^−^^3^
Monoclinic, *C*2/*c*	Mo *K*α radiation, λ = 0.71073 Å
Hall symbol: -C 2yc	Cell parameters from 3824 reflections
*a* = 21.795 (4) Å	θ = 2.6–27.4°
*b* = 10.077 (2) Å	µ = 5.64 mm^−^^1^
*c* = 29.967 (6) Å	*T* = 293 K
β = 110.67 (3)°	Block, yellow
*V* = 6158 (2) Å^3^	0.15 × 0.13 × 0.10 mm
*Z* = 4	

### Data collection

**Table:**

BRUKER SMART 1000 diffractometer	5995 independent reflections
Radiation source: fine-focus sealed tube	3563 reflections with *I* > 2σ(*I*)
graphite	*R*_int_ = 0.119
ω–scan	θ_max_ = 26.0°, θ_min_ = 3.4°
Absorption correction: multi-scan (*SADABS*; Sheldrick, 2004)	*h* = −26→26
*T*_min_ = 0.429, *T*_max_ = 0.569	*k* = −11→12
25098 measured reflections	*l* = −36→36

### Refinement

**Table:**

Refinement on *F*^2^	Secondary atom site location: difference Fourier map
Least-squares matrix: full	Hydrogen site location: inferred from neighbouring sites
*R*[*F*^2^ > 2σ(*F*^2^)] = 0.055	H-atom parameters constrained
*wR*(*F*^2^) = 0.151	*w* = 1/[σ^2^(*F*_o_^2^) + (0.0563*P*)^2^] where *P* = (*F*_o_^2^ + 2*F*_c_^2^)/3
*S* = 1.01	(Δ/σ)_max_ < 0.001
5995 reflections	Δρ_max_ = 1.09 e Å^−^^3^
317 parameters	Δρ_min_ = −1.11 e Å^−^^3^
0 restraints	Extinction correction: *SHELXL*, Fc^*^=kFc[1+0.001xFc^2^λ^3^/sin(2θ)]^-1/4^
Primary atom site location: structure-invariant direct methods	Extinction coefficient: 0.00036 (7)

### Special details

**Table:**

Geometry. All e.s.d.'s (except the e.s.d. in the dihedral angle between two l.s. planes) are estimated using the full covariance matrix. The cell e.s.d.'s are taken into account individually in the estimation of e.s.d.'s in distances, angles and torsion angles; correlations between e.s.d.'s in cell parameters are only used when they are defined by crystal symmetry. An approximate (isotropic) treatment of cell e.s.d.'s is used for estimating e.s.d.'s involving l.s. planes.
Refinement. Refinement of *F*^2^ against ALL reflections. The weighted *R*-factor *wR* and goodness of fit *S* are based on *F*^2^, conventional *R*-factors *R* are based on *F*, with *F* set to zero for negative *F*^2^. The threshold expression of *F*^2^ > σ(*F*^2^) is used only for calculating *R*-factors(gt) *etc*. and is not relevant to the choice of reflections for refinement. *R*-factors based on *F*^2^ are statistically about twice as large as those based on *F*, and *R*- factors based on ALL data will be even larger.

### Fractional atomic coordinates and isotropic or equivalent isotropic displacement parameters (Å^2^)

**Table:**

	*x*	*y*	*z*	*U*_iso_*/*U*_eq_	
Mo1	0.28396 (3)	0.20871 (5)	0.031782 (19)	0.0376 (2)	
N1	0.2149 (3)	0.1089 (5)	−0.07230 (18)	0.0397 (13)	
N2	0.2911 (3)	0.0167 (4)	−0.00225 (18)	0.0385 (13)	
N3	0.3695 (3)	0.2759 (5)	0.01561 (19)	0.0404 (13)	
N4	0.2950 (3)	0.3690 (5)	−0.05383 (18)	0.0405 (13)	
Br1	0.05852 (5)	0.01976 (10)	−0.28619 (3)	0.0768 (3)	
Br2	0.49853 (4)	−0.41859 (7)	0.08672 (3)	0.0659 (3)	
Br3	0.64761 (5)	0.06765 (10)	0.13147 (4)	0.0836 (3)	
Br4	0.23690 (6)	0.65632 (11)	−0.24584 (3)	0.0954 (4)	
C1	0.2551 (4)	0.0100 (6)	−0.0494 (2)	0.0425 (16)	
H1A	0.2583	−0.0652	−0.0664	0.051*	
C2	0.3574 (4)	0.3392 (6)	−0.0260 (2)	0.0438 (16)	
H2A	0.3919	0.3628	−0.0357	0.053*	
C11	0.1773 (3)	0.0879 (6)	−0.1214 (2)	0.0380 (15)	
C12	0.1655 (4)	0.1926 (7)	−0.1543 (2)	0.0535 (19)	
H12A	0.1805	0.2772	−0.1434	0.064*	
C13	0.1316 (4)	0.1730 (7)	−0.2030 (3)	0.061 (2)	
H13A	0.1260	0.2429	−0.2243	0.073*	
C14	0.1062 (4)	0.0477 (8)	−0.2194 (3)	0.057 (2)	
C15	0.1144 (4)	−0.0577 (7)	−0.1874 (3)	0.058 (2)	
H15A	0.0977	−0.1413	−0.1983	0.070*	
C16	0.1481 (4)	−0.0353 (7)	−0.1390 (3)	0.0530 (19)	
H16A	0.1515	−0.1041	−0.1176	0.064*	
C21	0.3353 (3)	−0.0897 (5)	0.0191 (2)	0.0360 (14)	
C22	0.3289 (4)	−0.2199 (6)	−0.0001 (2)	0.0482 (18)	
H22A	0.2927	−0.2411	−0.0268	0.058*	
C23	0.3763 (4)	−0.3170 (6)	0.0208 (3)	0.0543 (19)	
H23A	0.3713	−0.4021	0.0080	0.065*	
C24	0.4313 (4)	−0.2864 (6)	0.0608 (3)	0.0467 (17)	
C25	0.4366 (3)	−0.1634 (6)	0.0815 (2)	0.0451 (17)	
H25A	0.4722	−0.1442	0.1089	0.054*	
C26	0.3889 (4)	−0.0670 (6)	0.0615 (2)	0.0472 (18)	
H26A	0.3925	0.0147	0.0767	0.057*	
C31	0.4360 (3)	0.2377 (6)	0.0422 (2)	0.0401 (15)	
C32	0.4763 (4)	0.1811 (6)	0.0178 (2)	0.0462 (17)	
H32A	0.4607	0.1762	−0.0153	0.055*	
C33	0.5399 (4)	0.1330 (7)	0.0446 (3)	0.0512 (18)	
H33A	0.5662	0.0970	0.0291	0.061*	
C34	0.5629 (4)	0.1401 (7)	0.0945 (3)	0.0513 (18)	
C35	0.5243 (4)	0.1974 (6)	0.1187 (3)	0.0514 (19)	
H35A	0.5403	0.2040	0.1518	0.062*	
C36	0.4613 (4)	0.2443 (6)	0.0918 (2)	0.0490 (18)	
H36A	0.4357	0.2810	0.1077	0.059*	
C41	0.2845 (3)	0.4385 (6)	−0.0982 (2)	0.0406 (16)	
C42	0.2423 (4)	0.5467 (6)	−0.1095 (2)	0.0527 (19)	
H42A	0.2231	0.5759	−0.0881	0.063*	
C43	0.2285 (4)	0.6121 (7)	−0.1536 (3)	0.064 (2)	
H43A	0.2004	0.6848	−0.1612	0.077*	
C44	0.2570 (4)	0.5676 (7)	−0.1857 (3)	0.060 (2)	
C45	0.2992 (4)	0.4573 (7)	−0.1745 (3)	0.060 (2)	
H45A	0.3176	0.4271	−0.1963	0.072*	
C46	0.3135 (4)	0.3926 (7)	−0.1302 (2)	0.0509 (18)	
H46A	0.3419	0.3204	−0.1224	0.061*	

### Atomic displacement parameters (Å^2^)

**Table:**

	*U*^11^	*U*^22^	*U*^33^	*U*^12^	*U*^13^	*U*^23^
Mo1	0.0420 (4)	0.0429 (3)	0.0298 (3)	0.0039 (2)	0.0151 (3)	−0.0015 (2)
N1	0.042 (4)	0.046 (3)	0.034 (3)	0.001 (2)	0.016 (3)	−0.004 (2)
N2	0.042 (4)	0.039 (3)	0.037 (3)	0.003 (2)	0.016 (3)	−0.002 (2)
N3	0.035 (3)	0.049 (3)	0.039 (3)	0.004 (2)	0.015 (3)	−0.001 (2)
N4	0.045 (4)	0.046 (3)	0.031 (3)	0.006 (2)	0.014 (3)	0.006 (2)
Br1	0.0584 (6)	0.1298 (8)	0.0361 (5)	−0.0046 (5)	0.0089 (4)	−0.0152 (4)
Br2	0.0648 (6)	0.0618 (5)	0.0715 (6)	0.0196 (4)	0.0245 (5)	0.0237 (4)
Br3	0.0522 (6)	0.1145 (8)	0.0780 (7)	0.0227 (5)	0.0152 (5)	0.0185 (5)
Br4	0.1135 (10)	0.1272 (8)	0.0507 (6)	0.0062 (7)	0.0352 (6)	0.0316 (5)
C1	0.051 (5)	0.040 (3)	0.038 (4)	−0.001 (3)	0.017 (3)	−0.011 (3)
C2	0.052 (5)	0.045 (3)	0.035 (4)	0.005 (3)	0.017 (4)	0.000 (3)
C11	0.037 (4)	0.052 (4)	0.028 (3)	0.000 (3)	0.014 (3)	−0.009 (3)
C12	0.060 (5)	0.049 (4)	0.044 (4)	−0.008 (3)	0.010 (4)	0.000 (3)
C13	0.069 (6)	0.065 (5)	0.045 (4)	−0.011 (4)	0.017 (4)	0.006 (4)
C14	0.042 (5)	0.095 (6)	0.033 (4)	0.003 (4)	0.011 (4)	−0.005 (4)
C15	0.056 (6)	0.057 (4)	0.052 (5)	0.002 (4)	0.006 (4)	−0.012 (4)
C16	0.051 (5)	0.059 (4)	0.044 (4)	0.002 (3)	0.011 (4)	0.001 (3)
C21	0.035 (4)	0.042 (3)	0.033 (3)	0.003 (3)	0.015 (3)	0.000 (3)
C22	0.046 (5)	0.044 (4)	0.048 (4)	0.000 (3)	0.008 (4)	−0.003 (3)
C23	0.053 (5)	0.045 (4)	0.066 (5)	0.002 (3)	0.023 (4)	−0.005 (4)
C24	0.049 (5)	0.044 (4)	0.050 (4)	−0.003 (3)	0.022 (4)	0.010 (3)
C25	0.041 (5)	0.046 (4)	0.039 (4)	−0.004 (3)	0.003 (3)	−0.001 (3)
C26	0.057 (5)	0.038 (3)	0.044 (4)	−0.001 (3)	0.015 (4)	−0.004 (3)
C31	0.037 (4)	0.049 (4)	0.041 (4)	0.001 (3)	0.022 (3)	−0.001 (3)
C32	0.045 (5)	0.056 (4)	0.038 (4)	0.004 (3)	0.014 (4)	−0.002 (3)
C33	0.055 (5)	0.057 (4)	0.048 (4)	−0.005 (3)	0.026 (4)	0.000 (3)
C34	0.040 (5)	0.059 (4)	0.060 (5)	−0.002 (3)	0.023 (4)	0.002 (4)
C35	0.057 (5)	0.051 (4)	0.043 (4)	0.001 (3)	0.013 (4)	0.000 (3)
C36	0.052 (5)	0.050 (4)	0.048 (4)	0.004 (3)	0.021 (4)	−0.010 (3)
C41	0.040 (4)	0.052 (4)	0.033 (4)	−0.004 (3)	0.016 (3)	0.005 (3)
C42	0.065 (6)	0.058 (4)	0.041 (4)	0.006 (4)	0.028 (4)	0.002 (3)
C43	0.078 (7)	0.056 (4)	0.053 (5)	0.006 (4)	0.018 (5)	0.008 (4)
C44	0.071 (6)	0.064 (5)	0.042 (4)	−0.012 (4)	0.015 (4)	0.001 (4)
C45	0.072 (6)	0.073 (5)	0.049 (5)	−0.008 (4)	0.039 (5)	−0.009 (4)
C46	0.054 (5)	0.064 (4)	0.041 (4)	0.008 (4)	0.025 (4)	0.005 (3)

### Geometric parameters (Å, °)

**Table:**

Mo1—Mo1^i^	2.1263 (13)	C21—C26	1.410 (9)
Mo1—N3	2.192 (5)	C21—C22	1.419 (8)
Mo1—N4^i^	2.195 (5)	C22—C23	1.400 (9)
Mo1—N1^i^	2.198 (5)	C22—H22A	0.9300
Mo1—N2	2.218 (5)	C23—C24	1.398 (10)
N1—C1	1.345 (8)	C23—H23A	0.9300
N1—C11	1.424 (7)	C24—C25	1.372 (9)
N1—Mo1^i^	2.198 (5)	C25—C26	1.395 (9)
N2—C1	1.353 (8)	C25—H25A	0.9300
N2—C21	1.432 (7)	C26—H26A	0.9300
N3—C2	1.341 (8)	C31—C36	1.393 (9)
N3—C31	1.439 (8)	C31—C32	1.444 (9)
N4—C2	1.353 (8)	C32—C33	1.419 (9)
N4—C41	1.448 (7)	C32—H32A	0.9300
N4—Mo1^i^	2.195 (5)	C33—C34	1.401 (9)
Br1—C14	1.924 (7)	C33—H33A	0.9300
Br2—C24	1.929 (7)	C34—C35	1.413 (10)
Br3—C34	1.930 (7)	C35—C36	1.406 (9)
Br4—C44	1.919 (7)	C35—H35A	0.9300
C1—H1A	0.9300	C36—H36A	0.9300
C2—H2A	0.9300	C41—C42	1.389 (9)
C11—C12	1.404 (8)	C41—C46	1.402 (8)
C11—C16	1.410 (9)	C42—C43	1.411 (9)
C12—C13	1.398 (9)	C42—H42A	0.9300
C12—H12A	0.9300	C43—C44	1.390 (10)
C13—C14	1.396 (10)	C43—H43A	0.9300
C13—H13A	0.9300	C44—C45	1.405 (11)
C14—C15	1.399 (10)	C45—C46	1.412 (9)
C15—C16	1.393 (9)	C45—H45A	0.9300
C15—H15A	0.9300	C46—H46A	0.9300
C16—H16A	0.9300		
			
Mo1^i^—Mo1—N3	93.36 (14)	C23—C22—H22A	119.5
Mo1^i^—Mo1—N4^i^	92.14 (14)	C21—C22—H22A	119.5
N3—Mo1—N4^i^	174.47 (19)	C24—C23—C22	120.2 (6)
Mo1^i^—Mo1—N1^i^	92.07 (14)	C24—C23—H23A	119.9
N3—Mo1—N1^i^	91.09 (19)	C22—C23—H23A	119.9
N4^i^—Mo1—N1^i^	89.39 (19)	C25—C24—C23	119.8 (6)
Mo1^i^—Mo1—N2	93.96 (14)	C25—C24—Br2	120.7 (5)
N3—Mo1—N2	87.97 (18)	C23—C24—Br2	119.5 (5)
N4^i^—Mo1—N2	90.97 (18)	C24—C25—C26	120.3 (6)
N1^i^—Mo1—N2	173.9 (2)	C24—C25—H25A	119.9
C1—N1—C11	116.9 (5)	C26—C25—H25A	119.9
C1—N1—Mo1^i^	117.3 (4)	C25—C26—C21	121.9 (6)
C11—N1—Mo1^i^	125.8 (4)	C25—C26—H26A	119.0
C1—N2—C21	118.6 (5)	C21—C26—H26A	119.0
C1—N2—Mo1	114.5 (4)	C36—C31—N3	121.4 (6)
C21—N2—Mo1	126.4 (4)	C36—C31—C32	118.4 (6)
C2—N3—C31	118.2 (6)	N3—C31—C32	120.0 (6)
C2—N3—Mo1	116.6 (5)	C33—C32—C31	119.7 (6)
C31—N3—Mo1	124.4 (4)	C33—C32—H32A	120.1
C2—N4—C41	118.2 (6)	C31—C32—H32A	120.1
C2—N4—Mo1^i^	117.3 (4)	C34—C33—C32	119.7 (7)
C41—N4—Mo1^i^	124.2 (4)	C34—C33—H33A	120.1
N1—C1—N2	122.0 (5)	C32—C33—H33A	120.1
N1—C1—H1A	119.0	C33—C34—C35	121.0 (7)
N2—C1—H1A	119.0	C33—C34—Br3	120.1 (6)
N3—C2—N4	120.3 (6)	C35—C34—Br3	118.8 (6)
N3—C2—H2A	119.9	C36—C35—C34	118.7 (7)
N4—C2—H2A	119.9	C36—C35—H35A	120.6
C12—C11—C16	116.8 (6)	C34—C35—H35A	120.6
C12—C11—N1	120.7 (6)	C31—C36—C35	122.3 (7)
C16—C11—N1	122.5 (6)	C31—C36—H36A	118.9
C13—C12—C11	121.7 (6)	C35—C36—H36A	118.9
C13—C12—H12A	119.2	C42—C41—C46	120.8 (6)
C11—C12—H12A	119.2	C42—C41—N4	118.7 (6)
C14—C13—C12	119.5 (7)	C46—C41—N4	120.3 (6)
C14—C13—H13A	120.2	C41—C42—C43	119.9 (6)
C12—C13—H13A	120.2	C41—C42—H42A	120.0
C13—C14—C15	120.5 (7)	C43—C42—H42A	120.0
C13—C14—Br1	120.0 (6)	C44—C43—C42	119.8 (7)
C15—C14—Br1	119.5 (6)	C44—C43—H43A	120.1
C16—C15—C14	118.8 (7)	C42—C43—H43A	120.1
C16—C15—H15A	120.6	C43—C44—C45	120.4 (7)
C14—C15—H15A	120.6	C43—C44—Br4	119.3 (6)
C15—C16—C11	122.4 (7)	C45—C44—Br4	120.3 (6)
C15—C16—H16A	118.8	C44—C45—C46	119.9 (7)
C11—C16—H16A	118.8	C44—C45—H45A	120.1
C26—C21—C22	116.5 (6)	C46—C45—H45A	120.1
C26—C21—N2	119.6 (5)	C41—C46—C45	119.2 (6)
C22—C21—N2	123.9 (6)	C41—C46—H46A	120.4
C23—C22—C21	120.9 (7)	C45—C46—H46A	120.4

Symmetry codes: (i) −*x*+1/2, −*y*+1/2, −*z*.
